# Epidemiology and antimicrobial resistance of staphylococci other than *Staphylococcus aureus* from domestic animals and livestock in Africa: a systematic review

**DOI:** 10.3389/fvets.2022.1059054

**Published:** 2022-12-13

**Authors:** Remous Ocloo, Justin Nyasinga, Zubair Munshi, Aisha Hamdy, Tessa Marciniak, Manonmani Soundararajan, Mae Newton-Foot, Wilma Ziebuhr, Adebayo Shittu, Gunturu Revathi, Alaa Abouelfetouh, Andrew Whitelaw

**Affiliations:** ^1^Division of Medical Microbiology and Immunology, Stellenbosch University, Stellenbosch, South Africa; ^2^Department of Pathology, Aga Khan University Hospital, Nairobi, Kenya; ^3^Institute of Science, Technology and Innovation, Pan African University, Nairobi, Kenya; ^4^Department of Biomedical Sciences and Technology, The Technical University of Kenya, Nairobi, Kenya; ^5^Department of Microbiology and Immunology, Faculty of Pharmacy, Alexandria University, Alexandria, Egypt; ^6^Institute for Molecular Infection Biology, University of Wuerzburg, Wuerzburg, Germany; ^7^National Health Laboratory Service, Tygerberg Hospital, Cape Town, South Africa; ^8^Department of Microbiology, Obafemi Awolowo University, Ile-Ife, Nigeria; ^9^Department of Microbiology and Immunology, Faculty of Pharmacy, Alamein International University, Alamein, Egypt

**Keywords:** Africa, animals, antibiotic resistance, coagulase-negative staphylococci, non-aureus staphylococci, Staphylococci other than *S. aureus*

## Abstract

**Introduction:**

Staphylococci other than Staphylococcus aureus (SOSA) in animals are becoming more pathogenic and antibiotic resistant and can potentially disseminate to humans. However, there is little synthesized information regarding SOSA from animals in Africa. This systematic review provides a comprehensive overview of the epidemiology and antimicrobial resistance of SOSA in companion animals (pets) and livestock in Africa.

**Method:**

This systematic review (PROSPERO-CRD42021252303) was conducted according to the PRISMA guidelines, and 75 eligible studies from 13 countries were identified until August 2022. Three electronic databases (Pubmed, Scopus and Web of Science) were employed.

**Results:**

The frequently isolated SOSA were *S. epidermidis, S. intermedius, S. pseudintermedius, S. xylosus, S. chromogenes, S. hyicus, M. sciuri, S. hominis*, and *S. haemolyticus*. Thirty (40%) studies performed antibiotic susceptibility testing (AST). Penicillin (58%) and tetracycline (28%) resistance were most common across all SOSA with high rates of resistance to aminoglycosides, fluoroquinolones, and macrolides in some species. Resistance to last-resort antibiotics such as linezolid and fusidic acid were also reported. Limited data on strain typing and molecular resistance mechanisms precluded analysis of the clonal diversity of SOSA on the continent.

**Conclusion:**

The findings of this review indicate that research on livestock-associated SOSA in Africa is lacking in some regions such as Central and Western Africa, furthermore, research on companion animals and more advanced methods for identification and strain typing of SOSA need to be encouraged.

**Systematic review registration:**

https://www.crd.york.ac.uk/prospero/, identifier: CRD42021252303.

## Introduction

Staphylococci colonize the skin and mucous membranes of a wide range of vertebrate hosts and account for several human and animal diseases (1–4). Staphylococci other than *Staphylococcus aureus* (SOSA) is a relatively new term, encompassing largely coagulase-negative staphylococci, including two coagulase-positive staphylococcal species (*S. pseudintermedius* and *S. schleiferi*) (2). There are increasing reports of SOSA infections associated with implantable foreign bodies and sepsis, particularly among newborns and preterm neonates (5). Moreover, some SOSA (e.g., *S. pseudintermedius, S. schleiferi*, and *S. felis*) are important pathogens in veterinary medicine (6). SOSA infections are a global economic problem in the animal production sector (7–11). SOSA are largely responsible for mastitis in milk-producing animals, leading to reduced milk production (12).

Antimicrobials are administered to animals to enhance growth and prevent infection. However, the doses of antimicrobials dispensed by practitioners in animal husbandry are mostly sub-therapeutic and contribute to the emergence of antimicrobial resistance (AMR) (13–15). The spread of AMR genes may be exacerbated by trading with animals between farms and other animal husbandry practices (16, 17).

High resistance rates have been described in SOSA from animals. Tetracycline resistance has been described in SOSA isolates from normal and subclinical mastitic buffalo milk in Egypt: 100% in *S. lugdunensis* and *S. hominis*, and 66.6% in *S. epidermidis* (18). SOSA isolates recovered from turkey farms in Egypt were also resistant to tetracycline (100%), oxacillin (92.3%) and daptomycin (89.7%) (19). In Turkey, 75% of SOSA recovered from raw milk were resistant to erythromycin (20) and in South Africa, 51% of SOSA isolates from subclinical mastitis cow milk samples were multidrug resistant (MDR) (21). Antimicrobial-resistant SOSA (AMRSOSA) in livestock and companion animals pose an economic threat to the livestock industry and a risk for spill-over into the human environment, threatening public health (22–24).

SOSA are considered less pathogenic than *S. aureus* and this perception has led to limited knowledge on the virulence, prevalence, and dissemination of SOSA, particularly in animals (25, 26). However, in recent years, resistance to last-resort antibiotics has been described in SOSA (19, 27, 28). There is also evidence that SOSA and *S. aureus* exchange virulence and resistance genes continuously in the natural environment, which is a potential risk to the empiric treatment of *S. aureus* infections (29–33). For example, *Mammaliicoccus sciuri*, formerly known as *Staphylococcus sciuri*, is postulated to be the origin of *mecA* and the precursor to the evolution of the staphylococcal cassette chromosome *mec* (SCC*mec*) element (34, 35). The methicillin resistance gene (*mecA*) encodes an alternative penicillin-binding protein (PBP2A) (36, 37), which confers resistance to the β-lactam class of antibiotics such as penicillins and cephalosporins.

Systematic reviews on *S. aureus* in animals have observed that both animal clonal complex (CC) 398, CC130, CC133 and human-associated lineages (CC1, CC15, CC72, CC80, CC10, and CC152) are common in Africa. Methicillin resistant *S. aureus* (MRSA) prevalence ranges from 0 to 3 % (38, 39). However, the epidemiology of SOSA in companion animals and livestock in Africa is not well-described, despite the potential for transfer of staphylococci and their resistance mechanisms from these animals to humans. This study aims to provide a comprehensive overview of the published data describing the epidemiology and antimicrobial resistance of SOSA in companion animals and livestock in Africa.

## Methods

This systematic review was registered on https://www.crd.york.ac.uk/prospero/ (PROSPERO-CRD42021252303) and conducted following the preferred reporting items for systematic reviews and meta-analyses (PRISMA) guidelines (40).

### Literature search strategies

A literature search of relevant articles in PubMed, Scopus, and Web of Science, published from 1991 to 2022, was conducted and retrieved until August 2022 using the search terms described in Supplementary material 1. The results were transferred onto Microsoft Excel, and duplicates were removed. Titles and abstracts were screened, and full-text articles were assessed for eligibility. All searches and screenings were done by two independent reviewers each, and discrepancies were resolved by consensus.

### Eligible article selection

#### Inclusion criteria

Eligible studies included reports on SOSA isolated from companion animals and livestock in Africa and published in peer-reviewed English journals. Other criteria for inclusion were descriptions of population size and laboratory methods. *Mammaliicoccus sciuri* was included in this study as it was previously regarded as *S. sciuri*.

#### Exclusion criteria

Studies not published in English journals, published in predatory journals (according to Beall's list, 2021), which did not report on primary data, and those which reported on *S. aureus* only or did not perform speciation of SOSA were excluded. Review articles, notes, e-mails, editorials, articles without original data and studies that only described animal products and wild animals were also excluded.

### Data extraction and synthesis

The following data were extracted onto Microsoft Excel: first author, year of publication, study design, country/region, type of animals, number of animals, type of samples, number of samples, and year of sample collection. Other information included species isolated, number of isolates, susceptibility data for various classes of antibiotics (fluoroquinolones, cephalosporins, penicillins, aminoglycosides, macrolides, and polymyxins), resistance genes, laboratory methods, and strain types. Data extraction was performed by two independent reviewers and discrepancies were resolved by consensus. The data were stratified using the United Nations geoscheme subregion classification (https://unstats.un.org/unsd/methodology/m49/).

### Data interpretation

Antibiotic resistance rates were only reported for a species when a minimum of 30 isolates were tested (41). Antibiotic resistance rates for each species were calculated using the number of resistant isolates (NR) and total number of isolates tested (NT).


$$\begin{array}{l} \left(antibiotic\;resistance\;rate=\;\frac{NR}{NT}\right). \end{array}$$

Differences between antibiotic rates in carriage and pathogenic SOSA were calculated using Fisher exact and Chi squared tests where appropriate.

## Results

### Study description

**Figure 1:**
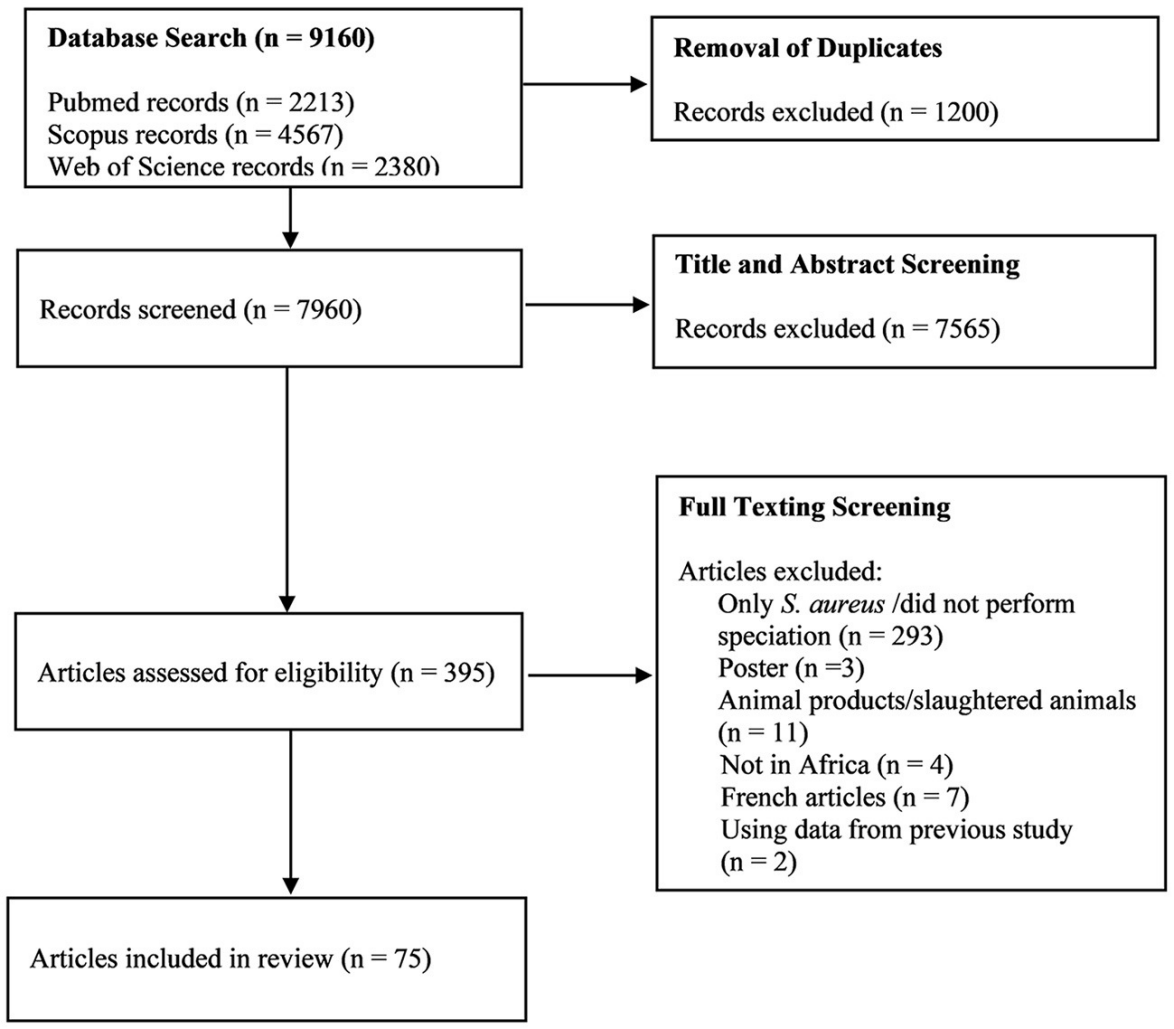
PRISMA flow diagram of the detailed search process and study selection.

**Figure 2:**
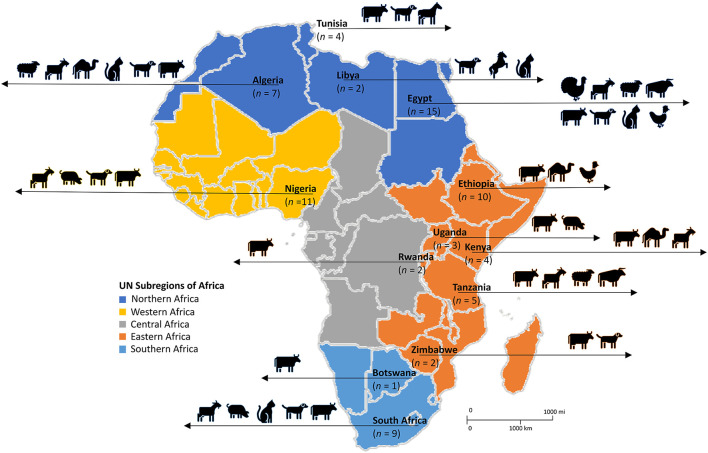
Diversity of livestock and domestic animals in which SOSA was described in Africa. Adapted from the United Nations geoscheme subregions African Map. *n*, number of articles.

The systematic search of the three databases yielded 9,160 articles. After de-duplication and exclusion by title and abstract screening, 395 full-text articles were screened, of which 75 were considered eligible based on our inclusion criteria (Figure 1). The majority of the studies (97%; *n* = 73), were published between 2000 and 2022, with 69% of the studies (*n* = 52) published between 2012 and 2022. According to regions, Northern Africa (*n* = 28) had the highest number of eligible studies, followed by Eastern Africa (*n* = 26), Western Africa (*n* = 11), and Southern Africa (*n* = 10) (Figure 2). No articles were available from Central Africa.

Of the 75 eligible studies, 62 described SOSA in livestock, 12 in companion animals, and one included both livestock and companion animal. Of the reports from livestock, 17 were performed on subsistence farms, nine on commercial farms, and the rest (*n* = 49) did not provide information on farm type. Overall, 46 reports were from diseased animals, 17 from healthy animals, and ten from diseased and healthy animals; while 2 did not provide information. Dogs were the most common companion animal investigated, in 12 studies, while cows and goats were the main livestock investigated, in 32 and 13 studies, respectively. Figure 2 represents the diversity of animals in which SOSA were described in Africa. Milk was the leading sample type screened, representing 65% (*n* = 49) of the eligible studies. Most SOSA were isolated from cows with mastitis (Table 1).

**Table 1:** Sources, clinical significance & geographical distribution of Staphylococci other than *S. aureus* (SOSA) in Africa.

**Country**	**Type of animal**	**Number of animals**	**Sampled population**	**Year of sample collection**	**Type of sample**	**Number of samples**	**Species (number of isolates)**	**Clinical significance**	**References**
**Eastern Africa**									
Ethiopia	Camel	348	Livestock	2010–2011	Milk	1,362	*S. hyicus* (27), *S. intermedius* (12)	Infection (clinical)	(42)
Ethiopia	Camel	253	Livestock	NR	Milk	956	*S. hyicus* (56), *S. epidermidis* (52)*, S. intermedius* (5)	Infection (clinical)	(43)
Ethiopia	Camel	96	Livestock	2015–2016	Milk	384	*S. hyicus* (3), *S. intermedius* (3)	Infection (clinical)	(44)
Ethiopia	Chicken	101	Livestock	NR	Cloacal Swabs	101	*S. hyicus* (8), *S. intermedius* (3)	Colonization	(45)
Ethiopia	Cow	186	Livestock	1997–1998	Milk	396	*S. epidermidis* (13)	Infection (clinical)	(46)
Ethiopia	Cow	NR	Livestock	NR	Milk	NR	*S. epidermidis* (35), *S. hyicus* (35), *S. intermedius* (5)	Infection (clinical)	(47)
Ethiopia	Cow	500	Livestock	NR	Milk	1,955	*S. epidermidis* (44), *S. intermedius* (4)	Infection (clinical)	(48)
Ethiopia	Cow	307	Livestock	1996–1997	Milk	1,133	*S. epidermidis* (2), *S. hyicus* (1)	Infection (clinical)	(49)
Ethiopia	Cow	404	Livestock	2016–2017	Milk	1,528	*S. lentus* (8), *S. sciuri* (2)	Infection (clinical)	(50)
Ethiopia	Cow	144	Livestock	2018–2019	Milk	576	*S. hyicus* (5), *S. intermedius* (2)	Infection	(51)
Kenya	Camel	206	Livestock	2017	Milk	798	*S. simulans* (12), *S. chromogenes* (5), *S. rostri* (5), *S. hyicus* (2), *S. delphini* (2) *S. epidermidis* (1), *S. haemolyticus* (1),	Both	(52)
Kenya	Camel	95	Livestock	2012	Milk	380	*S. epidermidis* (13)	Both	(53)
Kenya	Goat	110	Livestock	2019	Milk	80	*S. epidermidis* (1)	Infection (clinical)	(54)
Kenya	Goat	110	Livestock	NR	Milk	220	*S. intermedius* (1)	Infection (clinical)	(55)
Rwanda	Cow	256	Livestock	2016	Milk	418	*S. epidermidis* (46), *S. sciuri* (23), *S. chromogenes* (9), *S. xylosus* (9), *S. pasteuri* (8), *S. haemolyticus* (8), *S. capitis* (8)*, S. saprophyticus* (4), *S. devriesei* (2), *S. kloosii* (1), *S. lugdunensis* (1), *S. warneri* (1)	Infection (clinical)	(56)
Rwanda	Cow	112	Livestock	2018	Milk	303	*S. xylosus* (36), *S. haemolyticus* (24), *S. sciuri* (14), *S. chromogenes* (10), *S. saprophyticus* (9), *S. epidermidis* (8), *S. succinus* (5), *S. capitis* (3), *S. hominis* (2), *S. devriesei* (2), *S. auricularis* (2), *S. equorum* (2), *S. simulans* (1)	Infection (clinical)	(57)
Tanzania	Cow	NR	Livestock	2002	Milk	NR	*S. epidermidis* (55), *S. intermedius* (1)	Infection (clinical)	(58)
Tanzania	Cow	240	Livestock	2001	Milk	919	*S. epidermidis* (26), *S. intermedius* (10), *S. saprophyticus* (3), *S. hyicus* (1)	Infection (clinical)	(59)
Tanzania	Cow	416	Livestock	2014	Milk	1,648	*S. epidermidis* (134), *S. haemolyticus* (1)	Both	(60)
Tanzania	Cow	1,365	Livestock	1971–2002	Milk	1,964	*S. epidermidis* (32)	Infection (clinical)	(61)
Tanzania	Goat	43	Livestock	2004–2005	Milk	85	*S. epidermidis* (4)	Both	(62)
Uganda	Cow	97	Livestock	2010–2011	Milk	97	*S. saprophyticus* (4), *S. hyicus* (4), *S. xylosus* (3), *S. sciuri* (2), *S. epidermidis* (1), *S. hominis* (1), *S. haemolyticus* (1), *S. pasteuri* (1), *S. intermedius* (1), *S. gallinarum* (1), *S. lugdunensis* (1)	Infection (clinical)	(63)
Uganda	Cow	78	Livestock	NR	Milk	166	*S. epidermidis* (17), *S. haemolyticus* (3)	Infection (clinical)	(64)
Uganda	Pig	83	Livestock	2018–2019	Ear, nose, perine-um swabs	50	*S. simulans* (5), *S. cohnii* (2), *S. chromogenes* (2), *S. sciuri* (2), *S. lentus* (1), *S. petrasii* (1), *S. epidermidis* (1), *S. hyicus* (1)	Colonization	(65)
Zimbabwe	Cow	NR	Livestock	NR	Milk	406	*S. chromogenes* (32), *S. epidermidis* (30), *S. hominis* (24), *S. hyicus* (11), *S. xylosus* (9), *S. saprophyticus* (5), *S. lentus* (4), *S. sciuri* (2), *S. caseolyticus* (2), *S. simulans* (1), *S. muscae* (1), *S. kloosii* (1)	Infection (clinical)	(66)
Zimbabwe	Dog	NR	Domestic	1989–1990	Wound, skin & mouth swabs	87 85 39	*S. intermedius* (37)	Infection (clinical)	(67)
**Northern Africa**									
Algeria	Camel	17	Livestock	2014–2015	Milk	153	*S. arlettae* (10), *S. muscae* (8), *S. epidermidis* (5), *S. saccharolyticus* (5), *S. cohnii* (4), *S. succinus* (3), *S. saprophyticus* (2), *S. auricularis* (1), *S. capitis* (1), *S. hyicus* (6), *S. intermedius* (3)	Infection (clinical)	(68)
Algeria	Cat Dog	35 35	Domestic	2018–2019	Oral swabs	35 35	*S. xylosus* (28), *S. simulans* (10), *S. sciuri* (8), *S. saprophyticus* (7), *S. pseudintermedius* (6), *S. pseudointermedius/delphini/intermedius* (SIG) (5), *S. capitis* (2), *S cohnii-cohnii* (1), *S. epidermidis* (1)	Colonization	(69)
Algeria	Cow	NR	Livestock	NR	Milk	22	*S. xylosus* (12), *S. epidermidis* (4), *S. sciuri* (2), *S. lugdunensis* (2), *S. simulans* (1), *S. capitis* (1)	Infection (clinical)	(70)
Algeria	Cow	NR	Livestock	NR	Milk	NA	*S. hominis* (4), S. *haemolyticus* (2), *S. cohnii* (1), *S. xylosus* (1), *S. equorum* (1)	Infection (clinical)	(71)
Algeria	Cow	50	Livestock	NR	Milk	50	*S. xylosus* (16), *S. lentus* (5), *S. hominis* (1), *S*. *epidermidis* (1)	Infection (clinical)	(72)
Algeria	Goat	845	Livestock	2015–2018	Milk	815	*S. caprae* (18), *S. xylosus* (8), *S. simulans* (7), *S. epidermidis* (5)*, S. cohnii* (4), *S. lentus* (2), *S. hominis* (1)	Infection (clinical)	(73)
Algeria	Sheep	105	Livestock	2011–2012	Milk	105	*S. xylosus* (9), *S. epidermidis* (5), *S. lentus* (2)	Colonization	(74)
Egypt	Buffalo	NR	Livestock	NR	Milk	81	*S. intermedius* (11)*, S. xylosus* (7)*, S. epidermidis* (3), *S. hominis* (3), *S. sciuri* (1), *S. lugdunensis* (1), *S. simulans* (1), *S. hyicus* (1).	Both	(18)
Egypt	Buffalo Cow	50	Livestock	2018–2019	Milk	50	*S. warneri* (9), *S. pasteuri* (8), *S. xylosus* (4), *S. epidermidis* (2), *S. chromogenes* (2), *S. cohnii* (1), *S. hyicus* (1), *S. haemolyticus* (1), *S. sciuri* (1), *S. lentus* (1)	Infection (clinical)	(75)
Egypt	Buffalo Cow	14 53	Livestock	NR	Milk	68	*S. sciuri* (37), *S. chromogenes* (14), *S. haemolyticus* (10), *S. xylosus* (10), *S. hyicus* (2), *S. warneri* (1)	Infection (clinical)	(76)
Egypt	Buffalo Cow Goat Sheep	43 158 20 20	Livestock	NR	Milk	172 632 40 40	*S. xylosus* (68), *S. hominis* (49), *S. lugdunensis* (26), *S. cohnii* (26), *S. saprophyticus* (9), *S. chromogenes* (4), *S. lentus* (4), *S. simulans* (4), *S. haemolyticus* (1)	Both	(9)
Egypt	Cow	270	Livestock	2020	Milk	488	*S. xylosus* (64), *S. chromogenes* (23), *S. epidermidis* (22), *S. saprophyticus* (20), *S. haemolyticus* (18), *S. cohnii* (14), *S. simulans* (11), *S. hominis* (6), *S. lentus* (3).	Infection (clinical)	(77)
Egypt	Cow Sheep	100 25	Livestock	2019	Milk Abscess Swabs	100 25	*S. schleiferi* (29), *S. intermedius* (10), *S. xylosus* (3), *S. haemolyticus* (2), *S. epidermidis* (2)	Infection (clinical)	(78)
Egypt	Dog	NR	Domestic	NR	Ear Swabs	100	*S. pseudintermedius* (36)	Colonization	(79)
Egypt	Goat Sheep	44 20	Livestock	NR	Swabs	64	*S. epidermidis* (2)	Infection (clinical)	(80)
Egypt	Buffalo Cow	338 48	Livestock	NR	Milk	386	*S. intermedius* (30), *S. xylosus* (28), *S. hominis* (12), *S. epidermis* (10), *S. hyicus* (8), *S. chromogenes* (4), *S. caprae* (2), *S. simulans* (2), *S. lentus* (2), *S. lugdunensis* (2), *S. sciuri* (2)	Colonization	(81)
Egypt	Buffalo Cow	170 70	Livestock	NR	Milk	240	*S. carnosus* (14), *S. capitis* (10), *S. xylosus* (4), *S. saccharolyticus* (1), *S. auricularis* (1), *S. intermedius* (1)	Colonization	(82)
Egypt	Cat Cow Dog Goat Sheep	36 24 31 32 29	Both	NR	Nasal swabs	152	*S. felis* (2), *S. epidermidis* (1), *S. warneri* (1)	Both	(83)
Egypt	Cow	444	Livestock	NR	Milk	1,145	*S. chromogenes* (77)	Infection (clinical)	(84)
Egypt	Chicken	12	Livestock	NR	Synovial fluid	12	*S. epidermidis* (1), *S. lentus* (1), *S. hyicus* (1)	Infection (clinical)	(85)
Egypt	Goat Sheep	100 89	Livestock	2016–2017	Milk	289	*S. epidermidis* (4)	Infection (clinical)	(86)
Egypt	Turkey	NR	Livestock	2018	Cloacal swabs	250	*S. lentus* (16), *S. xylosus* (8), *S. saprophyticus* (5), *S. sciuri* (3), *S. condimenti* (2), *S. cohnii* (2), *S. simulans* (1), *S. epidermidis* (1), *S. arlettae* (1)	Colonization	(19)
Libya	Cat	NR	Domestic	NR	Nasal swab	103 48	*S. felis* (12), *S. sciuri* (8), *S. intermedius* (6), *S. capitis* (2), *S. cohnii* (1), *S. lentus* (1)	Both	(87)
Libya	Horse	92	Livestock	2018	Nasal swab	184	*S. xylosus* (12), *S. sciuri* (8), *S. equorum* (8), *S. lentus* (5), *S. simulans* (5), *S. gallinarum* (5), *S. chromogens* (4), *S. saprophyticus* (3), *S. intermedius* (3), *S. felis* (2), *S. warneri* (2), *S. pasteuri* (2), *S. haemolyticus* (2), *S. schleiferi* (2), *S. carnosus* (1), *S. kloosi* (1)	Colonization	(88)
Tunisia	Cow	NR	Livestock	2015–2016	Milk	112	*S. equorum* (9), *S. sciuri* (5), *S. xylosus* (4), *S. saprophyticus* (1), *S. cohnii* (1)	Infection (clinical)	(89)
Tunisia	Cow	300	Livestock	2013–2014	Milk	300	*S. xylosus* (27), *S. warneri* (8), *S. chromogenes* (6), *S. sciuri* (5), *S. epidermidis* (5), *S. pasteuri* (5), *S. haemolyticus* (4), *S. succinus* (3), *S. equorum* (2), *S. saprophyticus* (2), *S. cohnii* (1)	Infection (clinical)	(90)
Tunisia	Dog	100	Domestic	2011	Nasal swabs	100	*S. pseudintermedius* (55)	Colonization	(91)
Tunisia	Donkey	100	Livestock	2011–2012	Nasal swabs	100	*S. delphini* (19), *S. pseudintermedius* (2)	Colonization	(92)
**Southern Africa**									
Botswana	Cow	NR	Livestock	NR	Milk	NR	*S. xylosus* (27), *S. hyicus* (24)*, S. saprophyticus* (23), *S. sciuri* (17)*, S. epidermidis* (12), *S. lugdunensis* (11), *S. lentus* (11), *S. hominis* (8), *S. cohnii* (7), *S. haemolyticus* (6), *S. chromogenes* (4), *S. capitis* (3), *S. auricularis* (2), *S. simulans* (2)	Colonization	(93)
South Africa	Cat	NR	Domestic	2007–2012	Urine (fluid) Ear & skin swabs	216	*S. pseudintermedius/delphini/intermedius* (SIG) (17), *S. felis* (2), *S. simulans* (2)	Infection (clinical)	(94)
South Africa	Cow	1,374	Livestock	2013–2014	Milk	3,387	*S. chromogens* (80), *S. xylosus* (5), *S. hyicus* (4), *S. simulans* (4)*, S. haemolyticus* (2), *S. epidermidis* (1), *S. lugdunensis* (1).	Infection (clinical)	(95)
South Africa	Cow	NR	Livestock	2012	Milk	217	*S. xylosus* (19), *S. hominis* (13), *S. haemolyticus* (10), *S. sciuri* (9), *S. warneri* (8), *S. chromogenes* (5), *S. epidermidis* (4), *S. auricularis* (4), *S. cohnii- cohnii* (3), *S. cohnii- urealyticus* (2), *S. saprophyticus* (2), *S. hyicus* (1)	NR	(96)
South Africa	Cow	NR	Livestock	NR	Milk	NR	*S. chromogenes* (100), *S. epidermidis* (17), *S. haemolyticus* (16), *S. simulans* (3), *S. xylosus* (3), *S. hominis* (1), *S. sciuri* (1), *S. hyicus* (1)	Infection (clinical)	(21)
South Africa	Cow	384	Livestock	NR	Milk	384	*S. xylosus* (33), *S. chromogenes* (27), *S. hominis* (24), *S. warneri* (24), *S. sciuri* (6), *S. epidermidis* (6), *S. hyicus* (3), *S. saprophyticus* (3)	Colonization	(97)
South Africa	Cow Goat Pig	NR	Livestock	NR	Nasal & ear swabs Mouth wash	150	*S. haemolyticus* (42), *S. capitis* (18)*, S. xylosus* (18)	Colonization	(98)
South Africa	Dog	NR	Domestic	2007–2012	NA	334	*S. pseudintermedius* (278)	Infection (clinical)	(99)
South Africa	Dog	NR	Domestic	2007–2010	Skin, Pustule Skin swabs Skin biopsies Skin abscess and pustule swabs Fine needle aspirates	319	*S. intermedius* (319)	Infection (clinical)	(100)
South Africa	Dog	64	Domestic	2017–2019	Skin & Ear Swab	49	*S. pseudintermedius* (57), *S. epidermidis* (2)	Infection (clinical)	(101)
**Western Africa**									
Nigeria	Cow	211	Livestock	NR	Lesions	211	*S. epidermidis* (53)	Infection (clinical)	(102)
Nigeria	Dog	60	Domestic	1999–2000	Nasal Swabs	106	*S. epidermidis* (62)	Colonization	(103)
Nigeria	Dog	109	Domestic	NR	Swab	NR	*S. sciuri subspecies rodentium* (10), *S. lentus* (3), *S. haemolyticus* (2), *S. simulans* (1)	Colonization	(104)
Nigeria	Dog	NR	Domestic	NR	Wound Swabs	133	*S. epidermidis* (18)	Infection (clinical)	(105)
Nigeria	Goat	250	Livestock	NR	Milk	493	*S. epidermidis* (12), *S. chromogenes* (9), *S. caprae* (5), *S. auricularis* (4), *S. xylosus* (2), *S. lentus* (1)	Infection (clinical)	(106)
Nigeria	Goat	NR	Livestock	NR	Nasal swab	40	*S. epidermidis* (10)	Infection (clinical)	(107)
Nigeria	Goat	35	Livestock	1998–1999	Milk	35	*S. epidermidis* (27)	Infection (clinical)	(108)
Nigeria	Goat	101	Livestock	NR	Milk	202	*S. epidermidis* (4)	Both	(109)
Nigeria	Pig	291	Livestock	NR	Nasal swabs Ear swabs	NR	*S. sciuri* (10), *S. lentus* (6), *S. cohnii* (3), *S. haemolyticus* (1)	Colonization	(110)
Nigeria	Pig	120	Livestock	NR	Nasal swab	154	*S. sciuri* (2)*, S. warneri* (2), *S. xylosus* (1), *S. cohnii* (1)	Colonization	(111)
Nigeria	Pig	300	Livestock	2019	Nasal Swab	300	*S. haemolyticus* (19), *S. sciuri* (14), *S. intermedius* (11), *S. xylosus* (8), *S. simulans* (7), *S. schleiferi* (5), S. *schleiferi coagulans* (3), *S. hyicus* (3), *S. cohnii* (3), *S. lugdunensis* (3), *S. lentus* (3), *S. epidermidis* (2), *S. warneri* (2),	Colonization	(112)

NR, Not reported.

### Distribution of SOSA

**Figure 3:**
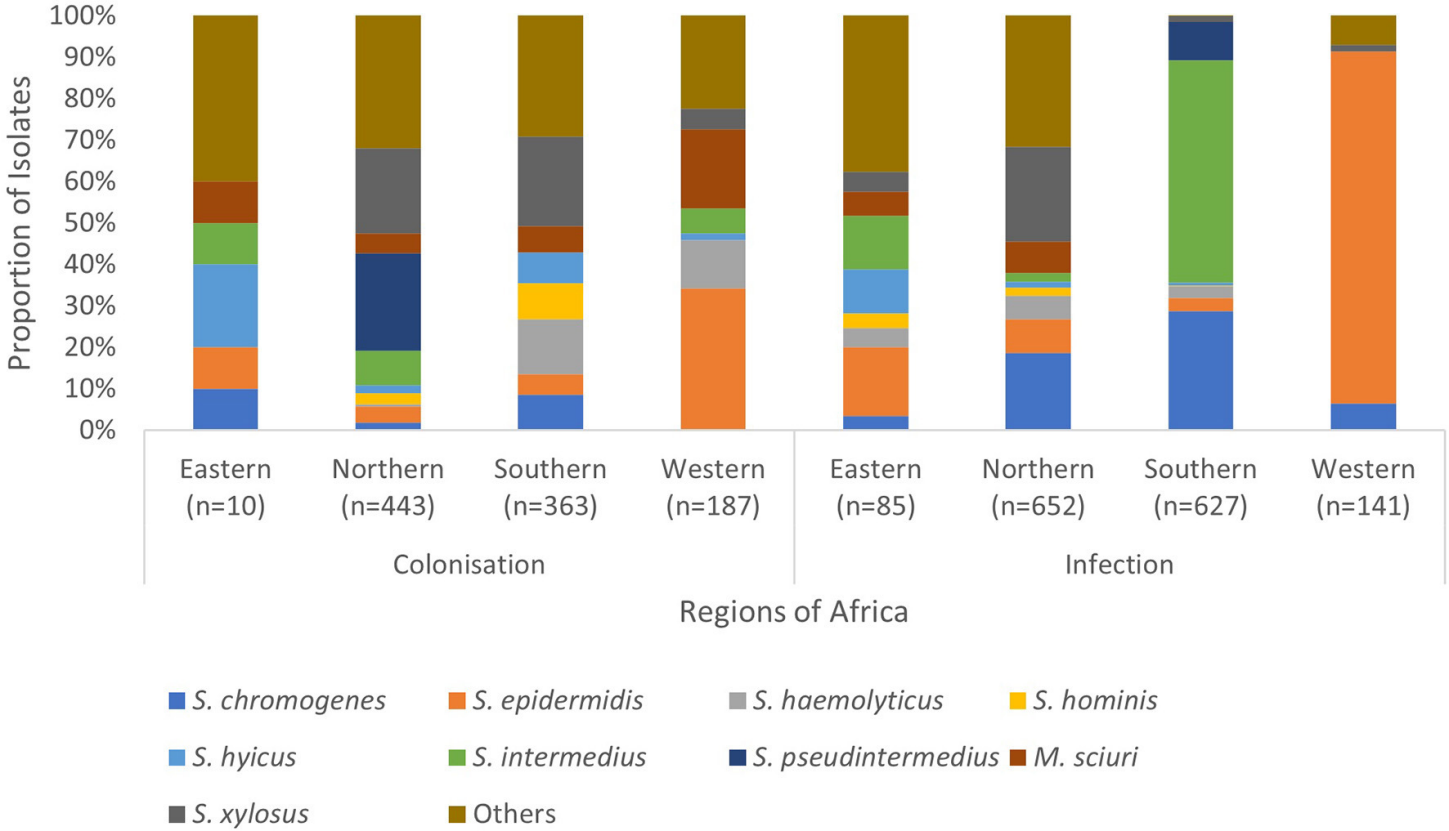
Regional distribution of SOSA in Africa. SOSA isolates were classified as “Others” when the total number included in the review was <100. Isolates (*n* = 177) in which colonization and infection were not distinguished were excluded. Eastern Africa (Ethiopia, Kenya, Rwanda, Tanzania, Uganda & Zimbabwe), Northern Africa (Algeria, Egypt, Libya & Tunisia), Southern Africa (Botswana & South Africa), Western Africa (Nigeria).

The frequently isolated SOSA in Africa were *S. epidermidis* (23%; *n* = 784), *S. intermedius* (14%; *n* = 446), *S. pseudintermedius* (11%; *n* = 371), *S. xylosus* (10%; *n* = 346), *S. chromogenes* (9.0%; *n* = 303), *S. hyicus* (4.7%; *n* = 157), *M. sciuri* (4.5%; *n* = 151), *S. hominis* (3.9%; *n* = 130), and *S. haemolyticus* (3.7%; *n* = 123) (Figure 3). *S. xylosus* was most frequently reported in Northern Africa, *S. intermedius*/*S*. *pseudintermedius* in Southern Africa, while *S. epidermidis* was predominant in Eastern and Western Africa (Table 1). API-Staph and BD Phoenix were the common biochemical tests employed to identify SOSA, in 64% (*n* = 48) studies (Supplementary material 2).

### Antibiotic susceptibility of SOSA

Thirty (40%) studies identified SOSA to the species level and performed antibiotic susceptibility testing (AST), of these only five (13%) were published prior to 2012. The Kirby Bauer disc diffusion method was widely utilized for AST (87%; *n* = 26) and the zone of inhibition was mostly interpreted using Clinical and Laboratory Standards Institute (CLSI) guidelines (81%; *n* = 21) (Supplementary material 2a).

**Figure 4:**
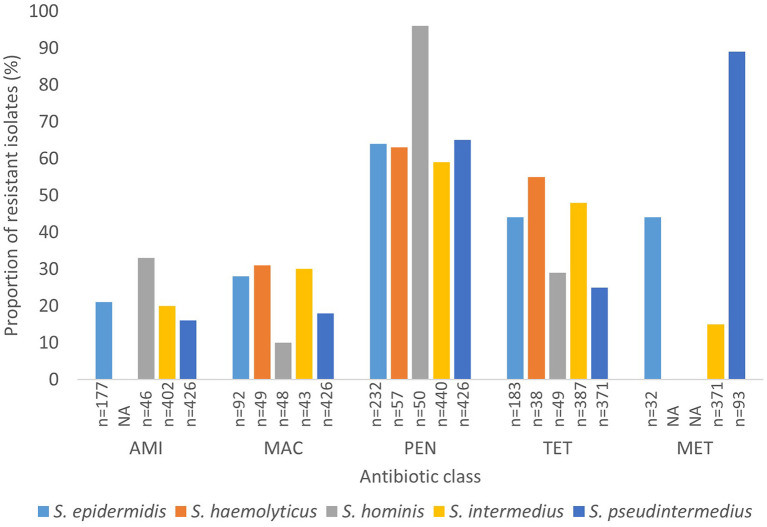
Rate of antibiotic resistance of clinically significant SOSA. AMI, Aminoglycoside; MAC, Macrolide; PEN, Penicillin; TET, Tetracycline; MET, Methicillin. “n” Denotes the number of isolates tested against a particular antibiotic. “NA” denotes not applicable.

Generally, data on methicillin resistance in SOSA was lacking. However, methicillin resistance rates were high among *S. pseudintermedius* (89%; *n* = 83) and *M. sciuri* (54%; *n* = 37), *S. xylosus* (45%; *n* = 37), and *S. epidermidis* (44%; *n* = 14) isolates. The typical human-associated SOSA showed high rates of penicillin resistance, *S. epidermidis* (64%), *S. haemolyticus* (63%), and *S. hominis* (96%), however, there was less resistance to glycopeptides and rifampicin. High rates of fluoroquinolone resistance were also observed in *S. epidermidis* (69%), *S. hyicus* (56%), *S. xylosus* (42%), and *M. sciuri* (36%). Aminoglycoside resistance was high in *S. xylosus* (31%), *M. sciuri* (36%), and *S. hominis* (33%) compared to the other SOSA. Lincosamide resistance was also high in *M. sciuri* (54%) and *S. xylosus* (31%) and *M. sciuri* demonstrated high rates of macrolide resistance (56%; *n* = 32). Overall, in SOSA, resistance to penicillin (58%) and tetracycline (28%) were most common (Supplementary material 2b). A few studies (*n* = 5) reported on susceptibility to last-resort antibiotics. Linezolid resistance was described in *S. intermedius* (*n* = 5), *S. xylosus* (*n* = 3), *S. equorum* (*n* = 1) and *S. epidermidis* (*n* =1) and resistance to fusidic acid was observed in *S. pseudintermedius* (*n* = 4), *M. sciuri* (*n* = 5), *S. xylosus* (*n* = 3), and *S. hominis* (*n* = 1) (Supplementary material 3). Figure 4 shows the rate of antibiotic resistance in SOSA species commonly encountered in the clinical setting. However, we were unable to stratify data geographically due to insufficient data from other regions.

Higher rates of aminoglycoside, fluoroquinolone, lincosamide, macrolide, rifampicin and methicillin resistance have been seen in carriage SOSA compared to pathogenic SOSA (Supplementary material 4).

### Antibiotic resistance mechanisms and strain typing

Fifteen (20%) studies performed molecular screening for AMR genes and reported the presence of the *mecA* gene in at least one member of the SOSA. The *mecA* was detected in *S. pseudintermedius* (*n* = 49), *S. sciuri* (*n* = 31), *S. intermedius* (*n* = 27), *S. xylosus* (*n* = 23), *S. epidermidis* (*n* = 12), *S. schleiferi* (*n* = 10), *S. haemolyticus* (*n* = 8), *S. hominis* (*n* = 3), *S. hyicus* (*n* = 2), and *S. chromogenes* (*n* = 1) (Supplementary material 5). Only three studies reported on SCC*mec* typing: SCC*mec* type V was noted in *S. warneri*, and SCC*mec* types I and IVa were detected in *S. epidermidis*. Only one study reported on sequence types (STs), diverse STs were described in *S. delphini* and *S. pseudintermedius* (Supplementary material 6).

## Discussion

Diverse SOSA were recovered from clinical and non-clinical samples from livestock and companion animals in Africa. The most common species include *S. epidermidis, S. pseudintermedius, S. xylosus*, and *S. chromogenes*. *S. pseudintermedius* was most commonly isolated in pets and *S. epidermidis* in livestock. However, there is lack of data on certain species in some countries such Botswana, Nigeria and Libya. We recommend that studies focus more on the following SOSA organisms which can also help understand the burden of SOSA infections in animals in Africa: *S. intermedius, S. xylosus* and *S. chromogene*s. *S. pseudintermedius* is considered as typical zoonotic commensal organism in the ears and skin of dogs and birds (79, 91). It is responsible for skin and soft tissue infections in dogs (67, 100), however in humans it is predominantly associated with infected dog bites and bacteraemia (113, 114). Surprisingly, none of the dogs from Western Africa (Nigeria) harbored *S. pseudintermedius* (104, 105, 108). *S. xylosus* is ubiquitous bacterium which is naturally occurring in the soil, food and on surfaces (115–117). Some strains are associated with opportunistic infections in humans (118, 119). There were no livestock associated infections/colonisations due to *S. xylosus* reported in Botswana, Ethiopia, Libya or Tanzania (42, 46, 58–62, 87, 93). *S. chromogenes* is a major cause of mastitis and subclinical mastitis in animals as seen in this review (21, 56, 57, 66, 75, 76, 84, 90, 106). However, no *S. chromogenes* infections were recorded in Botswana, Algeria, Ethiopia, Libya and Tanzania (42, 46, 58–62, 64, 70, 73, 87, 93, 120). The strain distribution could not be described due to the insufficient published data, but we were able to describe epidemiology of SOSA in terms of species distribution.

We observed that about 50% of the eligible studies utilized semi-automated methods (API-Staph and BD Phoenix) based on biochemical reactions. However, speciation of SOSA through these methods is unreliable as various species exhibit similar characteristics, hence molecular and spectrometric methods such as polymerase chain reaction (PCR) and matrix-assisted laser desorption/ionization time-of-flight mass spectrometry (MALDI-TOF MS) have been advocated (121, 122). Investigators pay little attention to SOSA as they are often considered contaminants, leading to the lack of speciation and strain typing. Therefore, there is sparse genotyping data on members of the SOSA, and it is difficult to understand the clonal diversity of SOSA on the continent and we could not describe clonal relationships with human-associated isolates.

The use of antibiotics in animal production and its consequent impact on AMR is a major challenge globally, particularly in Africa (15, 123, 124). We observed high rates of penicillin (58%) and tetracycline (28%) resistance in SOSA generally. The high rates (≥50%) of lincosamide, macrolide, and methicillin resistance observed in *M. sciuri* are note-worthy. Genes mediating resistance to these antibiotics could be transferred *via* mobile genetic elements to pathogenic bacteria such as *S. aureus*, as has previously been described for the SCC*mec* element (125, 126). High resistance to aminoglycosides, macrolides, tetracyclines, and methicillin was also observed in SOSA species (15–89%) which are commonly encountered in human medicine, such as *S. epidermidis, S. haemolyticus, S. hominis, S. intermedius*, and *S. pseudintermedius*, which might be due to the overuse of clinical antibiotics, particularly penicillin and tetracycline, in veterinary medicine (15). This could result in empiric treatment failures in clinical settings since these antibiotics are commonly used in human medicine. This again highlights the need for policymakers to enforce regulations on the use of antibiotics in animal husbandry in Africa.

Resistance to last resort antibiotics such as linezolid was also reported. This raises the risk of antibiotic resistance transfer to commensal *S. aureus* in animals, in which low rates of resistance to methicillin and other antibiotics have generally been observed in Africa (0–3%) (39). Interactions at the human-animal interface also raise the risk of antibiotic resistance transfer to humans. Studies have demonstrated that SOSA are becoming more resistant than *S. aureus* in humans (127, 128), although rates of methicillin resistance across Africa vary widely, from 12 to over 80% (129). The lack of methicillin resistance detection seen in this review might be due to the challenges associated with using cefoxitin and oxacillin for methicillin resistance screening in SOSA as discussed by Yang et al. (130) and Humphries et al. (2). Although, *mecA* or PBP2a PCR detection is the gold standard for assessment of methicillin resistance in staphylococci, PBP2a phenotypic testing may be an efficient, labor- and cost-saving approach (130, 131). The uneven distribution of studies has made it difficult to compare antibiotic resistant rates across the different regions. Furthermore, the high rates of antibiotic resistance seen in carriage SOSA can be attributed to the small sample size and the differences in species distribution but needs to be investigated further.

The findings from this review revealed that the epidemiology of SOSA is described mainly in cows, with insufficient data on companion animals such as dogs and cats in Africa. The very heterogeneous nature of the livestock and companion animals sampled in different regions does make it difficult to draw firm conclusions about the geographic distribution of different species and AMR rates among SOSA. We suggest that other livestock and companion animals should be investigated in future studies to better understand the problem of AMR in animals on the continent. Studies in Eastern Africa are more focused on infected livestock. Ethiopia particularly is home to Africa's largest livestock population and human interaction with healthy livestock is more frequent (132). We recommend that research in this region focuses on healthy animals to help address the problem of zoonotic transmissions. There is also a lack of data particularly in Central and Western Africa. It is important that research in these regions of Africa is encouraged to help understand the burden of AMR in animals in Africa. Few studies performed strain typing and molecular screening of antibiotic resistance genes. With the decrease in the cost of next-generation sequencing, researchers in Africa should consider employing whole genome sequencing (WGS), which can also provide additional data such as resistance, virulence, pathogenicity, and genetic composition of the organism (133).

Many studies did not indicate the sample collection period, and there was inadequate longitudinal data, describing SOSA or AMR in specific countries or regions; for example, from West Africa only Nigeria reported on SOSA in animals. Therefore, it was not possible to stratify data which is a limitation of the review. However, findings from this review shows that SOSA species are becoming more resistant to antibiotics, and this requires immediate attention.

## Data availability statement

The original contributions presented in the study are included in the article/Supplementary material, further inquiries can be directed to the corresponding author/s.

## Author contributions

RO contributed to the conceptualization and design of the systematic review, performed literature searches, data extraction and synthesis, data interpretation, and wrote the manuscript. JN, ZM, AH, TM, and MS performed literature searches, data extraction and synthesis, and provided critical feedback on the manuscript. MN-F, AW, WZ, GR, AA, and AS contributed to the conceptualization and design of the systematic review and provided critical feedback on the manuscript. All authors contributed to the article and approved the submitted version.
