# Supplementary material for: Epidemiology and antimicrobial resistance of staphylococci other than Staphylococcus aureus from domestic animals and livestock in Africa: a systematic review
Source: Front Vet Sci. 2022 Dec 13;9:1059054. doi: 10.3389/fvets.2022.1059054 (PMC9792789; doi:10.3389/fvets.2022.1059054)
Supplement: Supplementary file 1 [file Table_1.DOCX]

**PubMed Advanced Search**

| **Search** | Query |
| --- | --- |
| 1 | ((((animal*) OR (veterinar*)) OR (bird*)) OR (livestock)) OR (poultry) |
| 2 | (staphylococc*) OR (*Mammaliicocc**) |
| 3 | (Algeria OR Angola OR Benin OR Botswana OR Burkina Faso OR “Burkina Faso” OR Burkina Fasso OR Upper Volta OR "Upper Volta" OR Burundi OR Cameroon OR Cape Verde OR "Cape Verde" OR Central African Republic OR Chad OR Comoros OR “Iles Comores” OR Iles Comores OR Comoro Islands OR “Comoro Islands” OR Congo OR Democratic Republic Congo OR “Democratic Republic of the Congo” OR Zaire OR Djibouti OR Egypt OR Equatorial Guinea OR "Equatorial Guinea" OR Eritrea OR Ethiopia OR Gabon OR Gambia OR Ghana OR Guinea OR Guinea Bissau OR "Guinea Bissau" OR Ivory Coast OR “Ivory Coast” OR Cote d'Ivoire OR "Cote d'Ivoire" OR Kenya OR Lesotho OR Liberia OR Libya OR Libia OR Jamahiriya OR Jamahiryia OR Madagascar OR Malawi OR Mali OR Mauritania OR Mauritius OR Ile Maurice OR “Ile Maurice” OR Morocco OR Mozambique OR Moçambique OR Namibia OR Niger OR Nigeria OR Rwanda OR Sao Tome OR "Sao Tome" OR Senegal OR Seychelles OR Sierra Leone OR "Sierra Leone" OR Somalia OR South Africa OR "South Africa" OR Sudan OR South Sudan OR “South Sudan” OR Swaziland OR Tanzania OR Tanganyika OR Zanzibar OR Togo OR Tunisia OR Uganda OR Western Sahara OR "Western Sahara" OR Zambia OR Zimbabwe OR Africa OR Africa* OR Southern Africa OR West Africa OR Western Africa OR Eastern Africa OR East Africa OR North Africa OR Northern Africa OR Central Africa OR Sub Saharan Africa OR Subsaharan Africa OR Sub-Saharan Africa) |
| 4 | #1 AND #2 AND #3 |
| 5 | #1 AND #2 AND #3 fillter |

**Web of Science Advanced Search**

| **Search** | Query |
| --- | --- |
| 1 | TS=(animal OR animals OR livestock OR poultry OR bird OR birds OR veterinary OR veterinaries) |
| 2 | TS=(*Staphylococcus* OR staphylococci OR *Mammaliicoccus* OR mammaliicocci) |
| 3 | TS=(Algeria OR Angola OR Benin OR Botswana OR Burkina Faso OR “Burkina Faso” OR Burkina Fasso OR Upper Volta OR "Upper Volta" OR Burundi OR Cameroon OR Cape Verde OR "Cape Verde" OR Central African Republic OR Chad OR Comoros OR “Iles Comores” OR Iles Comores OR Comoro Islands OR “Comoro Islands” OR Congo OR Democratic Republic Congo OR “Democratic Republic of the Congo” OR Zaire OR Djibouti OR Egypt OR Equatorial Guinea OR "Equatorial Guinea" OR Eritrea OR Ethiopia OR Gabon OR Gambia OR Ghana OR Guinea OR Guinea Bissau OR "Guinea Bissau" OR Ivory Coast OR “Ivory Coast” OR Cote d'Ivoire OR "Cote d'Ivoire" OR Kenya OR Lesotho OR Liberia OR Libya OR Libia OR Jamahiriya OR Jamahiryia OR Madagascar OR Malawi OR Mali OR Mauritania OR Mauritius OR Ile Maurice OR “Ile Maurice” OR Morocco OR Mozambique OR Moçambique OR Namibia OR Niger OR Nigeria OR Rwanda OR Sao Tome OR "Sao Tome" OR Senegal OR Seychelles OR Sierra Leone OR "Sierra Leone" OR Somalia OR South Africa OR "South Africa" OR Sudan OR South Sudan OR “South Sudan” OR Swaziland OR Tanzania OR Tanganyika OR Zanzibar OR Togo OR Tunisia OR Uganda OR Western Sahara OR "Western Sahara" OR Zambia OR Zimbabwe OR Africa OR Africa* OR Southern Africa OR West Africa OR Western Africa OR Eastern Africa OR East Africa OR North Africa OR Northern Africa OR Central Africa OR Sub Saharan Africa OR Subsaharan Africa OR Sub-Saharan Africa) |
| 4 | #1 AND #2 AND #3 |
| 5 | #1 AND #2 AND #3 filter |

**Scopus**

(*Staphylococcus* OR staphylococci OR *Mammaliicoccus* OR mammaliicocci) AND (animal OR animals OR livestock OR poultry OR bird OR birds OR veterinary OR veterinaries)

NB: Scopus search was limited to the countries in the Web of Science and PubMed advanced search
