# Supplementary material for: Epidemiology and antimicrobial resistance of staphylococci other than Staphylococcus aureus from domestic animals and livestock in Africa: a systematic review
Source: Front Vet Sci. 2022 Dec 13;9:1059054. doi: 10.3389/fvets.2022.1059054 (PMC9792789; doi:10.3389/fvets.2022.1059054)
Supplement: Supplementary file 2 [file Table_2.DOCX]

| Species | AMI | | FLU | | GLY | | LIN | | MAC | | PEN | | SUL | | TET | | CHL | | RIF | | MET | |
| --- | --- | --- | --- | --- | --- | --- | --- | --- | --- | --- | --- | --- | --- | --- | --- | --- | --- | --- | --- | --- | --- | --- |
|  | NT | NR  (%) | NT | NR  (%) | NT | NR  (%) | NT | NR  (%) | NT | NR  (%) | NT | NR  (%) | NT | NR  (%) | NT | NR  (%) | NT | NR  (%) | NT | NR  (%) | NT | NR  (%) |
| ***S. chromogenes*** | 122 | 5  (4) | 91 | 3  (3) | 35 | 1  (3) | 97 | 3  (3) | 128 | 9  (7) | 141 | 57  (40) | 93 | 1  (1) | 128 | 10  (8) | 36 | 2  (6) | 31 | 0  (0) | 85 | 1  (1) |
| ***S. epidermidis*** | 177 | 38  (21) | 58 | 40  (69) |  |  |  |  | 92 | 26  (28) | 232 | 149  (64) | 42 | 11  (26) | 183 | 80  (44) | 80 | 15  (19) |  |  | 32 | 14  (44) |
| ***S. haemolyticus*** |  |  |  |  |  |  |  |  | 49 | 15  (31) | 57 | 36  (63) | 34 | 7  (21) | 38 | 21  (55) | 43 | 10  (23) |  |  |  |  |
| ***S. hominis*** | 46 | 15  (33) |  |  | 50 | 4  (8) |  |  | 48 | 5  (10) | 50 | 48  (96) |  |  | 49 | 13  (27) | 46 | 13  (28) |  |  |  |  |
| ***S. hyicus*** | 77 | 4  (5) | 52 | 29  (56) |  |  |  |  | 68 | 12  (18) | 77 | 18  (23) |  |  | 77 | 10  (13) | 61 | 4  (7) |  |  | 39 | 5  (13) |
| ***S. intermedius*** | 402 | 80  (20) | 392 | 26  (7) | 52 | 14  (27) | 376 | 40  (11) | 43 | 13  (30) | 440 | 258  (59) | 360 | 88  (24) | 387 | 184  (48) | 335 | 5  (1) | 349 | 2  (1) | 371 | 57  (15) |
| ***S. pseud- intermedius*** | 426 | 69  (16) | 335 | 50  (15) |  |  | 420 | 144  (29) | 426 | 78  (18) | 426 | 277  (65) | 390 | 69  (18) | 371 | 94  (25) | 333 | 18  (5) | 36 | 2  (6) | 93 | 83  (89) |
| ***M. sciuri*** | 59 | 21  (36) | 42 | 15  (36) | 48 | 5  (10) | 37 | 20  (54) | 57 | 32  (56) | 98 | 65  (66) |  |  | 53 | 27  (51) |  |  |  |  | 68 | 37  (54) |
| ***S. xylosus*** | 130 | 40  (31) | 74 | 31  (42) | 96 | 24  (25) | 78 | 24  (31) | 109 | 32  (29) | 179 | 106  (59) | 54 | 9  (17) | 154 | 43  (28) | 86 | 24  (28) | 61 | 2  (3) | 82 | 37  (45) |
| **Total** |  | 272  (16) |  | 194  (11) |  | 48 (3) |  | 231  (13) |  | 222 (13) |  | 1014  (58) |  | 185  (11) |  | 482  (28) |  | 91  (5) |  | 6  (0.3) |  | 234  (13) |

NR = number SOSA species resistant, NT = number SOSA species tested, AMI = aminoglycoside, FLU = fluroquinolone,

GLY = glycopeptide, LIN = lincosamide, MAC = macrolide, PEN = penicillin, SUL = sulfonamides, TET = tetracycline, CHL = chloramphenicol, RIF = rifampicin, MET = methicillin.

Resistance rates were only reported for species when a minimum of 30 isolates were tested (Clinical and Laboratory Standards Institute).

Shaded cells: resistance was not reported, or less than 30 isolates were tested.

Antibiotic resistance rates were only reported for a species when a minimum of 30 isolates were tested (41). Antibiotic resistance rates for each species were calculated using the number resistant isolates (NR) and total number of isolates tested (NT).

($antibiotic resistance rate=\frac{NR}{NT}$).

Total number of SOSA tested=1745
